# Radiographic Knee Osteoarthritis Is a Risk Factor for the Development of Dementia: Locomotive Syndrome and Health Outcomes in the Aizu Cohort Study

**DOI:** 10.3390/jcm13164956

**Published:** 2024-08-22

**Authors:** Yuji Endo, Hiroshi Kobayashi, Kazuyuki Watanabe, Koji Otani, Kenichi Otoshi, Hironori Numazaki, Miho Sekiguchi, Mari Sato, Takuya Nikaido, Rei Ono, Shin-ichi Konno, Yoshihiro Matsumoto

**Affiliations:** 1 Department of Orthopedic Surgery, Fukushima Medical University School of Medicine, Fukushima 960-1295, Japan; yjed@fmu.ac.jp (Y.E.); kazu-w@fmu.ac.jp (K.W.);; 2 Department of Sports Medicine, Fukushima Medical University, Fukushima 960-1295, Japan; 3 Department of Rehabilitation Medicine, Fukushima Medical University School of Medicine, Fukushima 960-1295, Japan; 4 Department of Physical Activity Research, National Institute of Biomedical Innovation, Health, and Nutrition—National Institute of Health and Nutrition, Osaka 566-0002, Japan

**Keywords:** dementia, knee, osteoarthritis, prospective study

## Abstract

Objective: Osteoarthritis is linked to dementia, but no longitudinal studies have established this connection. This prospective cohort study from the Locomotive Syndrome and Health Outcome in Aizu Cohort Study (LOHAS) aimed to determine if knee osteoarthritis (KOA) independently predicts dementia in adults aged 65 and above. Methods: Participants were classified by the Kellgren–Laurence scale into no/minimal KOA (grades 0 and I) and definitive KOA (grade II or higher). We analyzed dementia incidence from 2009 to 2015 using long-term care insurance data, adjusting for age, sex, vascular risks, depressive symptoms, and activity levels. Results: Out of 1089 participants (58.9% female, average age 72.5), 72.0% had definitive KOA. Dementia occurrence was significantly higher in the definitive group (8.4%) compared to the no/minimal group (3.0%) (*p* < 0.001). A log-rank test and Cox regression analysis confirmed these findings, showing an adjusted hazard ratio of 2.29 (confidence interval: 1.12–4.68) for dementia in those with definitive KOA. Conclusions: These results suggest that KOA is a significant risk factor for dementia, highlighting the importance of addressing contributing factors in KOA patients to potentially slow the progression of dementia.

## 1. Introduction

As the aging population has continued to increase, 1 in 10 people worldwide were aged ≥65 years in 2021 [1]. The incidence of dementia has increased accordingly, with a systematic review showing that the pooled prevalence for all-cause dementia was 697 (95% confidence interval [CI]: 546–864) per 10,000 persons among individuals aged ≥50 years, with a higher burden in women [2,3]. In 2020, there were over 55 million people worldwide living with dementia; this number is projected to reach 78 million and 139 million in 2030 and 2050, respectively [4]. Globally, the cost of dementia was estimated to be USD 1.3 trillion in 2018 [5]. This cost is expected to increase exponentially as the number of people living with dementia is projected to triple by 2050 [5]; therefore, reducing the occurrence of this condition is imperative. In recent years, improving health habits has been recognized to play a significant role in preventing dementia, necessitating tailored strategies that suit the specific conditions of each country and region [6].

Knee osteoarthritis (KOA) is a degenerative musculoskeletal disorder characterized by joint deformity that manifests as cartilage deterioration and osteophyte development, leading to pain and compromised joint function in affected individuals. Risk factors that increase the likelihood of developing KOA include age, being female, overweight or obesity, and knee injuries, in addition to occupational factors such as frequent kneeling, heavy lifting, and squatting [7]. This pathology not only causes physical discomfort in patients but also limits their mobility, significantly affecting daily activities and their overall quality of life. Physical activity levels are diminished due to KOA, leading to the development of several lifestyle diseases and increasing mortality rates by approximately 20% [8]. Approximately 73% of people with osteoarthritis are older than 55 years, with a prevalence of 365 million. Osteoarthritis most commonly occurs in the knees, followed by the hips and hands [9]. Additionally, the global economic cost of osteoarthritis cannot be precisely estimated, although it represents a significant burden [10]. The number of patients with KOA is expected to increase as the world’s population ages [2,11].

Musculoskeletal disorders have been linked to the onset of dementia. For example, one cohort study has shown that lumbar spinal stenosis increases the incidence of dementia [12]. Meta-analyses have shown that osteoarthritis is significantly associated with dementia [13]. One theory proposes that local cytokines may negatively impact neuronal cells. Nevertheless, as these studies are not longitudinal, further research is needed to clarify whether osteoarthritis directly contributes to the development of dementia. Currently, longitudinal studies that definitively establish a causal relationship between KOA and the development of dementia are limited or lacking.

The initiation of KOA (typically occurring in the age range of 40 to 50 years [7]) precedes the onset of dementia, which generally manifests at 60 years of age or later [14]. Radiographic techniques have been extensively used to diagnose a variety of traumas and orthopedic disorders due to their ease of use. However, radiographic changes indicative of KOA may occur before clinical symptoms become apparent. Notably, radiographic changes in KOA do not consistently align with the presence of symptoms, particularly during the early stages of the condition [15]. We hypothesized that radiological KOA could lead to the development of dementia and ultimately decrease physical activity levels. The present study investigated the relationship between radiological KOA and the development of dementia in community-dwelling older adults using data from “the Locomotive Syndrome and Health Outcome in Aizu Cohort Study (LOHAS)”, a population-based prospective cohort study.

The primary objective of this study is to investigate the potential relationship between knee deformities and the onset of dementia. Given the increasing prevalence of both conditions in aging populations worldwide, understanding whether structural changes in the knee could influence cognitive decline is crucial. This research holds significance as it has the potential to uncover novel insights into the impact of musculoskeletal health on neurological conditions. By identifying possible links, our study could pave way for early interventions that not only preserve joint health but also protect cognitive function, ultimately improving the quality of life for elderly individuals.

## 2. Materials and Methods

### 2.1. Study Design

This study used data from LOHAS, a population-based prospective cohort study on the risk of cardiovascular disease, quality of life, medical costs, and mortality attributable to locomotor dysfunction. Additionally, LOHAS provides the epidemiological information required for policymaking regarding locomotor dysfunction detection. Participants in LOHAS were residents of Minamiaizu and Tadami in Fukushima Prefecture, Japan, who underwent regular health examinations conducted by the municipalities in 2009 who also opted for a locomotor health examination. Our examination was performed at the same time. The details of the study design were previously described [16]. Participants aged ≥65 years with bilateral frontal plain knee joint radiographs were included. Participants who had already undergone total knee arthroplasty or high tibial osteotomy and those with dementia at the time of participation were also excluded.

We also used data from Japan’s long-term care insurance certification (LTCI), which is officially submitted by each municipality and includes all participants who needed care, died, or moved away.

All subjects provided their written informed consent for the use of their collected data before they participated in the study. The study was conducted in accordance with the Declaration of Helsinki, and the protocol was approved by the Research Ethics Committee in our university in April 2008 (No. 673).

### 2.2. Classification of Participants

Participants with a Kellgren–Laurence (KL) classification [17] of grade 0 or I on a simple radiograph of the knee joint in the standing position were classified into the no/minimal KOA group, while those with grade II or higher were classified into the definitive KOA group. The bilateral sides were measured individually, and the grades were assigned by two board-certified orthopedic surgeons. In cases of disagreement, the grades were reassessed and finalized through mutual consultation. Moreover, in instances where the severity differed between sides, the more severe grade was designated as the patient’s overall grade. The intraclass correlation coefficient had an intra-rater reliability κ = 0.653 and an inter-rater reliability κ = 0.652 [18]. When the assessments of the KL grade by the two knee surgeons did not align, a consensus was achieved through deliberation. As the reported intra- and inter-observer reliabilities of the KL grade classification were 0.56 and 0.61, respectively, our grading accuracy was either equal to or surpassed that of a previous report [19].

### 2.3. Outcome Measures and Definition of Dementia

Dementia, as newly diagnosed by the attending physician, was class II or higher according to the criteria for dementia in the Japanese LTCI. Class II dementia was defined as follows: “They can be independent if someone pays attention to them, even if they show some symptoms/behaviors or communication difficulties that interfere with their daily lives” [20]. Using the official LTCI data, we comprehensively investigated the incidence of dementia among all community-dwelling older adults.

### 2.4. Measurement of Confounding Factors

Based on current evidence, we included age; sex; vascular risk factors such as diabetes, hypertension, and dyslipidemia; depressive symptoms; and daily activity. Diabetes mellitus was defined as a random glucose concentration of 200 mg/dL, fasting glucose concentration of 126 mg/dL, or HbA1c ≥ 6.5% or use of hypoglycemic medication. Hypertension was defined as systolic blood pressure > 130 mmHg, diastolic blood pressure > 85 mmHg, or use of antihypertensive medication. Dyslipidemia was defined as a fasting blood count of low-density lipoprotein cholesterol ≥ 140 mg/dL, high-density lipoprotein cholesterol < 40 mg/dL, triglycerides ≥ 150 mg/dL, or use of dyslipidemia medication.

Depressive symptoms were identified using the Center for Epidemiologic Studies Depression Scale [21] (CES-D), which consists of 20 items. A score of ≥10 was defined as the presence of depressive symptoms.

The International Physical Activity Questionnaire (IPAQ) [22] was employed to assess the daily activity levels of the participants. Individuals were categorized based on their activity levels: Those who engaged in moderate or higher levels of physical activity were classified as having a high level of daily activity. Conversely, individuals with less-than-moderate activity levels were classified as having a low level of daily activity. Moderate-intensity physical activity can be defined as any activity that induces light sweating which measures at 3.0 to 5.9 metabolic equivalents (METs) [23].

### 2.5. Statistical Analysis

All investigated factors were compared based on the KOA grade using the *t*-test and Chi-square test.

Demographic characteristics, main exposure (KL classification grade II or higher), and outcome (development of dementia) are described as appropriate indices. Multiple imputations by chained equations were used to handle missing covariate data. Five complete datasets were generated, each using covariates, and substituted with a set of valid values to replace missing values.

Kaplan–Meier curves were used to describe the time to onset of dementia among participants with no/minimal KOA and definitive KOA groups, and significant differences were examined in a log-rank test. The Cox proportional hazards model was used to describe the relationship between participant characteristics (including KL grade) and the development of dementia. Statistical analyses were performed using IBM SPSS Statistics ver.28.0.1.0 for Mac (IBV Japan Inc., Tokyo, Japan). Statistical significance was set at *p* < 0.05.

## 3. Results

### 3.1. Participant Selection

**Figure 1:**
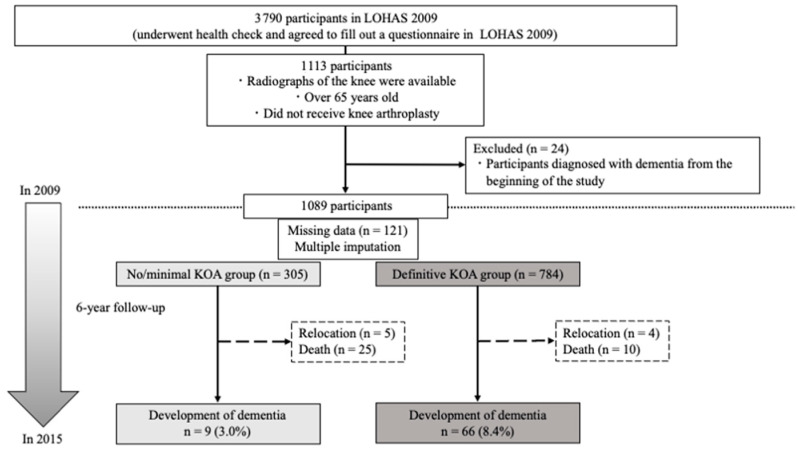
Flow chart of the study cohort from 2009 to 2015, showing initial participant numbers, exclusions, and outcomes. After exclusions for age, knee surgery, and pre-existing conditions, 1089 out of 1113 eligible participants were followed for 6 years, with data on dementia incidence and mortality reported. LOHAS: Locomotive Syndrome and Health Outcomes in the Aizu Cohort Study.

Of the total 3790 LOHAS participants in 2009, 1113 were eligible for inclusion, characterized by having available knee radiographs, being aged 65 years or older, and not having undergone knee arthroplasty. However, during initial assessments, 24 participants were excluded due to a diagnosis of dementia. Finally, 1089 participants were included in the final analysis (Figure 1).

### 3.2. Participant Baseline Characteristics

Table 1 summarizes the baseline characteristics of the participants. The mean age of the participants was 72.5 (±5.0) years; 58.9% were female. The definitive KOA group included 784 participants and the no/minimal KOA group included 305 participants. The definitive KOA group, which consisted of 72.0% of the patients with a mean age of 73.0 ± 4.2 years, included participants significantly older than those in the no/minimal group (mean age: 71.2 ± 4.2 years). Furthermore, the definitive KOA group had more female participants than males (63.3% vs. 47.5%, respectively), compared with the no/minimal KOA group (*p* < 0.001). However, there were no significant differences in the other parameters. By 2015, 9 participants relocated and 35 died, resulting in a follow-up rate of 96.0%.

### 3.3. Study Outcome: Dementia

All participants, regardless of their status in 2015, including death or relocation, were included in the final analysis. Among the 1089 participants, 75 (6.9%) developed dementia in 2015, including 66 (8.4%) and 9 (3.0%) participants in the definitive KOA and no/minimal KOA groups, respectively. The definitive KOA group showed a significantly higher rate of dementia development relative to the no/minimal KOA group (*p* < 0.001) (Figure 1).

### 3.4. Knee Osteoarthritis and Dementia

**Figure 2:**
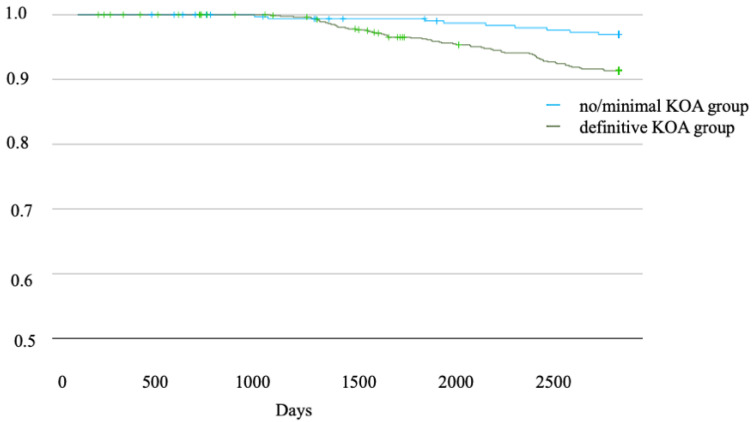
Kaplan–Meier curves illustrating the cumulative incidence of dementia development between the two groups, revealing a significant divergence, as evidenced by a log-rank test (*p* < 0.001). KOA: Knee osteoarthritis.

**Figure 3:**
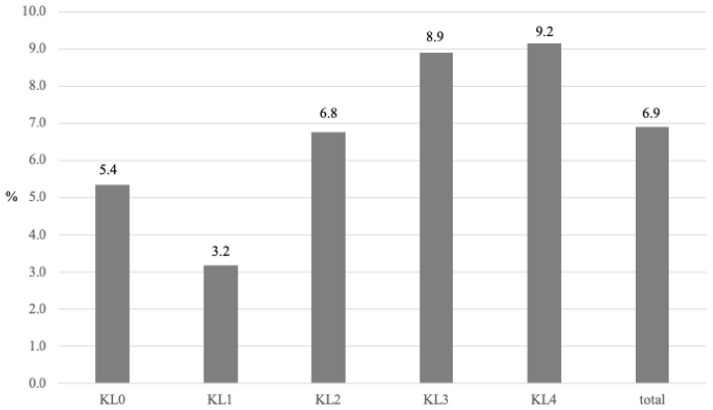
Incidence of dementia according to the KL classification. The incidence of dementia increases as the KL grade increases. KL: Kellgren–Laurence classification.

Over the six-year follow-up period, Kaplan–Meier curves were used to depict the cumulative incidence of dementia development between the two groups. The curves showed a significant divergence, supported by the log-rank test (*p* < 0.001) (Figure 2). In participants with definitive KOA, a higher KL grade was found to be associated with a higher incidence of dementia. Specifically, the incidence rates of dementia were 5.4% for KL grade 0, 3.2% for grade 1, 6.8% for grade 2, 8.9% for grade 3, and 9.2% for grade 4. These findings indicate that as the KL grade increases, the incidence of dementia increases correspondingly (Figure 3).

### 3.5. Risk Factors for Dementia

Cox regression analysis illustrated a heightened dementia development rate among the definitive KOA group compared to the no/minimal KOA group after adjusting for confounding factors (hazard ratio [HR] of 2.29, 95%CI; 1.12–4.68) (Table 2).

### 3.6. Analysis of Missing Data

Despite the presence of missing data in 121 cases (11.1%)—attributable to variables such as diabetes, hypertension, and dyslipidemia, which are considered vascular risk factors, along with CES-D score and daily activity level—an analysis using a dataset devoid of missing values yielded an HR of 2.12 (95% CI; 0.99–4.54). This result mirrors the original finding, thus reinforcing the consistency of the outcome despite the application of multiple imputation techniques to address data gaps (Table 3).

## 4. Discussion

To the best of our knowledge, this is the first longitudinal study to clarify the causal relationship between KOA and dementia. We found that the participants in the definitive KOA group had a significantly higher risk of developing dementia even after adjusting for age, sex, diabetes, hypertension, dyslipidemia, depressive symptoms, and daily activity level. Weber et al. propose that the mechanisms connecting osteoarthritis and dementia remain undefined as a result of insufficient research into their association [11]. While our study does not completely clarify the underlying mechanisms linking KOA and dementia, it contributes progressively to the existing body of knowledge by providing incremental insights into their relationship.

In recent years, the association between dementia and musculoskeletal diseases has been studied, and lumbar spinal stenosis has been reported as a risk factor for the development of dementia [10]. Weber et al. have reported a correlation between osteoarthritis and dementia [11]. Furthermore, exercise has been suggested to be useful in the prevention and treatment of dementia [24], and the link between musculoskeletal disorders and dementia has been extensively discussed. Multiple mechanisms have been suggested whereby KOA may precipitate dementia, with previous studies implicating secondary declines in activity levels [25,26] and lifestyle-related diseases [27,28,29,30] as confounding factors. In the present study, these variables were considered confounders. Nevertheless, KOA emerged as an independent risk factor for dementia, even after adjustment.

Chronic inflammation caused by osteoarthritis is believed to be a factor in dementia pathogenesis. Proinflammatory cytokines and chemokines that are upregulated in osteoarthritis may traverse the systemic circulation and blood–brain barrier, eliciting cerebral inflammation. This inflammatory state can facilitate neuronal damage and cell death, thereby impairing cognitive function. The persistent elevation of these inflammatory agents may foster neuroinflammation, leading to neurodegeneration [31].

Furthermore, many studies suggest that physical activity levels are associated with dementia risk. However, we did not find a direct association between dementia development and moderate or higher physical activity levels as measured by the IPAQ. Additionally, PGC-1α (a transcriptional coactivator pivotal to energy metabolism regulation in musculoskeletal conditions) is postulated to play a significant role. Physical activity induces PGC-1α upregulation, which augments mitochondrial function, boosts fatty acid metabolism, stimulates gluconeogenesis, and mitigates oxidative stress, collectively implying its potential in sustaining musculoskeletal health [32,33]. Animal studies suggest an association between PGC-1α level and dementia in mice [34,35,36]. Given its ties to energy metabolism, the PGC-1α level may mirror physical activity level. Whether bio-chemical factors such as PGC-1α level are directly related to the development of dementia is also a subject for future investigation.

This study may have a significant impact on outpatients. Recently, mild cognitive impairment (MCI) is drawing attention as a precursor state of dementia and is a very common condition with a prevalence rate of 5–30% [37]. A significant number of out-patients with KOA may also have MCI. Identifying intervening factors is important in patients with radiological KOA because it is difficult to improve the grade of radiological KOA through intervention [38]. Future studies should consider focusing on different geographic regions, extending the follow-up periods, and including confounding factors, such as diet and social activities, for a better understanding of the potential of appropriate KOA treatment in reducing the incidence of dementia.

### 4.1. Significance and Strengths of This Study

This was a prospective cohort study with a 6-year follow-up period. The greatest strength of this study is that it is one of the few longitudinal studies on this subject, as many similar studies are cross-sectional. Another strength is the use of an objective viewpoint (the frontal view of a plain radiograph of the knee joint) for the diagnosis of KOA. Third, there were few dropouts (only 18 participants; follow-up rate, 98.3%) because the study was conducted in a low-mobility area.

### 4.2. Limitations of This Study

Firstly, we used the definitions provided in the opinion forms submitted to the municipality for the certification of long-term care needs to comprehensively investigate the occurrence of dementia. This approach may have excluded individuals without long-term care insurance, potentially overlooking some dementia cases. Additionally, we did not use the International Statistical Classification of Diseases and Related Health Problems or Diagnostic and Statistical Manual of Mental Disorders scales, which are commonly used criteria to determine dementia. Assessing dementia from the perspective of care for older adults, which may be clinically relevant, could lack the rigor of established criteria. Secondarily, the study was conducted in a rural area. In this study, the prevalence of radiographic KOA was higher than that previously reported [39,40], possibly because our study participants lived in rural areas and their primary industry was agriculture [41]. This geographic specificity may have introduced certain biases. Therefore, for better generalization of the findings in this study, similar studies should be conducted in other areas where residents have different lifestyles and activities. Thirdly, subjective information regarding knee pain was not included in the analysis. Because it may be both a cause and a consequence of underlying conditions such as KOA, which complicates its role in cognitive decline, we considered knee pain as an intermediate factor, and knee pain was not used for adjustment in this study. We considered knee pain as an intermediate factor because it may be both a cause and a consequence of underlying conditions such as KOA, which complicates its role in cognitive decline [42]. Excluding knee pain from our analysis allowed us to focus directly on the effects of KOA progression independently of the subjective pain experiences, which can vary widely among individuals. Another limitation was the lack of information regarding KOA treatment during follow-up. In future studies, it would be beneficial to include investigations of specific treatments, such as injections or medication or rehabilitation, and to evaluate their effects. This approach could provide crucial data on the management and outcomes of KOA, which would enhance our understanding of effective interventions. Fourthly, the study did not adjust for factors related to diet, social activities, and education, which could also significantly influence the development of dementia. This decision was made based on giving preference to factors considered more clinically significant, according to the frequency of the outcomes observed.

In conclusion, radiographic KOA is identified as an independent risk factor for the development of dementia. Future research should scientifically clarify the relationship between musculoskeletal disorders and cognitive function, as well as investigate whether therapeutic interventions for KOAs can reduce the incidence of dementia.

## Figures and Tables

**Table 1:** Demographic data of participants in the no/minimal KOA group and definitive KOA group.

	Total *n* = 1089	No/Minimal KOA Group *n* = 305 (28.0%)	Definitive KOA Group *n* = 784 (72.0%)
Age (average ± SD)	72.5 ± 5.0	71.2 ± 4.2	73.0 ± 4.2 *
Sex (women)	641 (58.9%)	145 (47.5%)	498 (63.3%) *
Hypertension	800 (73.5%)	213 (69.8%)	587 (74.9%)
Diabetes	380 (34.9%)	104 (34.1%)	276 (35.2%)
Dyslipidemia	551 (50.6%)	151 (49.5%)	400 (51.0%)
CES-D ≥ 10	121 (11.1%)	30 (9.8%)	91 (11.6%)
Daily activity	432 (39.7%)	127 (41.6%)	305 (38.9%)

KOA: Knee osteoarthritis; SD: Standard deviation; CES-D: the Center for Epidemiologic Studies Depression scale; Daily activity: Defined by the rating chart devised using the International Physical Activity Questionnaire as “moderate-intensity walking or exercise at least once a week.” * *p* < 0.05.

**Table 2:** The Cox proportional hazard model adjusted for confounders by using multiple imputations for missing data.

	Number of Subjects	Development of Dementia	Occurrence Rate/Year	HR (CI)
no/minimal KOA group	305	9	0.49	Reference
Definitive KOA group	784	66	1.40	2.29 (1.12–4.68)

KOA: Knee osteoarthritis. HR: Hazard ratio. CI: Confidence interval.

**Table 3:** The Cox proportional hazards model adjusted for confounders in complete data.

	Number of Subjects	Development of Dementia	Occurrence Rate/Year	HR (CI)
no/minimal KOA group	280	5	0.30	Reference
definitive KOA group	688	46	1.11	2.12 (0.99–4.54)

KOA: Knee osteoarthritis. HR: Hazard ratio. CI: Confidence interval.

## Data Availability

No new data were created or analyzed in this study.
